# Interdisciplinary management of an impacted dilacerated maxillary central incisor

**DOI:** 10.1590/2177-6709.23.3.037-046.oar

**Published:** 2018

**Authors:** Harpreet Singh, Pranav Kapoor, Poonam Sharma, Pooja Dudeja, Raj Kumar Maurya, Surbhi Thakkar

**Affiliations:** 1 ESIC Dental College and Hospital, Department of Orthodontics and Craniofacial Orthopedics (New Delhi, India).; 2 ESIC Dental College and Hospital, Department of Conservative Dentistry and Endodontics (New Delhi, India).; 3 Army Dental Centre (New Delhi, India).; 4 Private Practice (New Delhi, India).

**Keywords:** Apicoectomy, Dilaceration, Impacted, Orthodontic traction, Surgical exposure

## Abstract

**Introduction:**

Tooth dilacerations are dental anomalies characterized by an abrupt deviation in the longitudinal axis of a tooth. They may occur either in the crown, between the crown and root, or in the root. Although not so common, impacted maxillary incisors exhibiting root dilaceration pose a diagnostic and treatment challenge to the clinician.

**Description:**

This case report describes the management of a horizontally impacted and dilacerated maxillary central incisor in a 12-year-old girl. Cone-beam computed tomographic scans were used to accurately localize the position of the dilacerated tooth, and to assess the extent of root formation and degree of dilaceration present in the root. Treatment included surgical exposure and orthodontic traction, followed by root canal treatment and apicoectomy.

**Results:**

Through a meticulously planned interdisciplinary approach, the impacted dilacerated central incisor was properly aligned and demonstrated good stability after the long-term follow-up.

**Conclusion:**

Taking into consideration the concerns and expectations of the patient, communicative feedback between the oral surgeon, orthodontist and endodontist helped achieving successful esthetic, structural and functional outcome in the present case.

## RESUMO

**Introdução:**

as dilacerações dentárias são anomalias caracterizadas por desvio acentuado no eixo longitudinal de um dente. Elas podem ocorrer na coroa, entre a coroa e a raiz, ou na raiz. Apesar de não serem muito comuns, os incisivos superiores impactados apresentando dilaceração radicular representam um desafio para o clínico, quanto ao diagnóstico e tratamento.

**Descrição:**

o presente relato de caso descreve o tratamento de um incisivo central superior impactado horizontalmente e com dilaceração, em uma menina com 12 anos de idade. Tomografias computadorizadas de feixe cônico foram utilizadas para localizar com precisão a posição do dente dilacerado e avaliar o grau de formação e de dilaceração da raiz. O tratamento incluiu exposição cirúrgica e tração ortodôntica, seguida de tratamento do canal radicular e apicectomia.

**Resultados:**

por meio de uma abordagem interdisciplinar meticulosamente planejada, o incisivo central impactado com dilaceração foi devidamente alinhado e demonstrou boa estabilidade em acompanhamento de longo prazo.

**Conclusão:**

levando-se em consideração as preocupações e expectativas da paciente, a comunicação interativa adotada entre o cirurgião oral, ortodontista e o endodontista ajudou na obtenção de resultados estéticos, estruturais e funcionais satisfatórios no presente caso.

## INTRODUCTION

Unerupted maxillary anterior teeth, by virtue of their position, have a major impact on dental and facial aesthetics and tend to adversely affect the nutritional and psychosocial well-being of the patient. One of the causes of permanent central incisor eruption failure is crown or root dilaceration. The term ‘dilaceration’ refers to a dental anomaly characterized by an abrupt deviation in the longitudinal axis of the root or crown of a formed tooth.^1^ Most frequently, it affects the root of a tooth, which may curve either in the labiolingual or mesiodistal direction.^2^ Since in a dilacerated tooth, the direction of the root is not in accordance with that of the crown, the normal eruptive pathway may be lost or the tooth may never even erupt. However, teeth with milder or more apical dilacerations may erupt spontaneously. 

Treatment modalities employed for impacted dilacerated maxillary incisors include surgical extraction accompanied by orthodontic space closure or fixed prosthesis,^3^ surgical repositioning^4^, autotransplantation,^5^ and forced eruption using a surgical-orthodontic approach.^6^ ^-^ ^11^

Orthodontic traction following surgical exposure, accompanied by endodontic therapy has proven to be a viable treatment option in aptly selected cases.^6^ ^,^ ^9^ Even so, an impacted incisor with a severely dilacerated root often poses a diagnostic, management and prognostic challenge to the clinician, due to the risks of ankyloses or ectopic eruption involved. Moreover, penetration of the labial cortical plate by the curved root, loss of attachment or external root resorption are other unfavorable sequalae that further compound the challenging disimpaction process.^11^ ^,^ ^12^ This article aims to report the well-synchronized orthodontic-surgical-endodontic management of a horizontally upward impacted and severely dilacerated maxillary central incisor.

## CASE PRESENTATION

A 12-year-old girl presented seeking treatment for unerupted permanent right maxillary central incisor. She reported very low self-esteem as she was constantly bullied due to her unesthetic smile. The patient was otherwise physically healthy and had no history of any medical illness. Neither the parents nor the patient could recall any history of trauma to the teeth or jaws.

## DIAGNOSIS AND TREATMENT PLANNING

**Figure 1:**
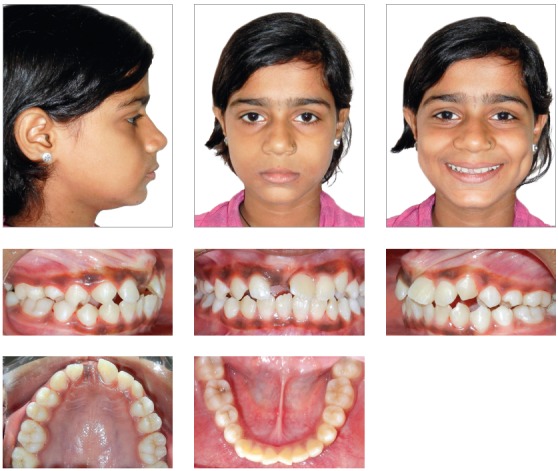
Pretreatment photographs.

The patient had a skeletal Class I jaw-base relationship and a mesofacial pattern. Intraoral examination revealed that all permanent teeth were in the state of partial or complete eruption, except for the maxillary right central incisor. She displayed Class I molar and Class II canine relationship, with an overbite of 2 mm and an overjet of 2 mm. Mild crowding in maxillary and mandibular arches was observed. The clinical absence of the right maxillary central incisor and the mesiolabial rotation of left maxillary central incisor resulted in an inadequate space distribution (Fig 1). The crown of the unerupted incisor was palpable as a labial bulge high in the vestibular sulcus. 

**Figure 2:**
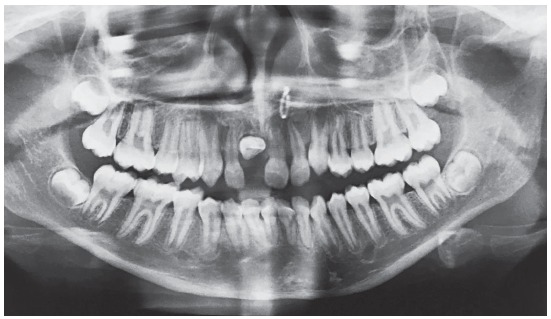
Pretreatment panoramic radiograph showing the horizontally impacted maxillary incisor.

**Figure 3:**
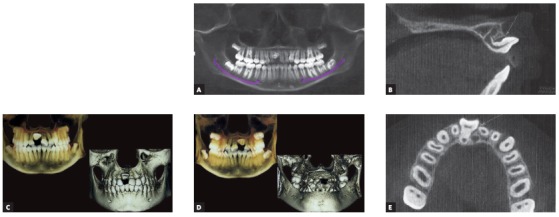
A) 3-D reformation: Tru-Pan view. B) Pretreatment CBCT image with 3-D reconstruction in sagittal plane, showing severely dilacerated root of the impacted incisor with a crown-root angle of 100^o^. C) 3-D reconstruction - labial view. D) 3-D reformation - lingual view. E) 3-D reconstruction - axial plane.

Panoramic radiograph demonstrated an impacted right maxillary central incisor without clear visualization of root morphology (Fig 2).The three-dimensional cone-beam computerized tomographic (CBCT) reconstructed images showed that the long axis of the horizontally impacted incisor was oriented buccopalatally, and the labial aspect was positioned higher up in the alveolus, with the palatal aspect facing towards and above the alveolar crest. The impacted incisor had approximately three-fourth of normal root formation, with a crown-root angle of 100^o^, and superiorly directed dilaceration in apical aspect of the root. Cervical aspect of the tooth crown was in close proximity to the nasopalatine foramen, and apical aspect of the root was abutting the root of right maxillary lateral incisor (Fig 3). 

Possible treatment options ranging from autotransplantation, surgical extraction of the involved tooth accompanied by fixed prosthesis, and surgical exposure followed by orthodontic traction were discussed. Since the root of the impacted incisor was severely dilacerated, some degree of difficulty would be expected during extraction and subsequent replantation, along with the impending risk of ankylosis. Hence autotransplantation was not considered to be a viable treatment option. Also, considering the relatively young age of the patient, extraction of the incisor followed by permanent prosthodontic rehabilitation at a much later stage following growth cessation was deemed impractical due to the chances of alveolar bone loss in the extraction site during the waiting period. Moreover, since the patient and her parents were very keen on saving the incisor, it was decided to surgically expose the impacted tooth followed by orthodontic treatment to align it into the arch, at the same time maintaining the integrity of the supporting periodontal tissues. Considering the possible risks of root resorption and perforation of labial cortical plate, the patient and her parents were informed regarding the ensuing need for root canal treatment and apicoectomy during treatment. 

## TREATMENT PROGRESS

**Figure 4:**
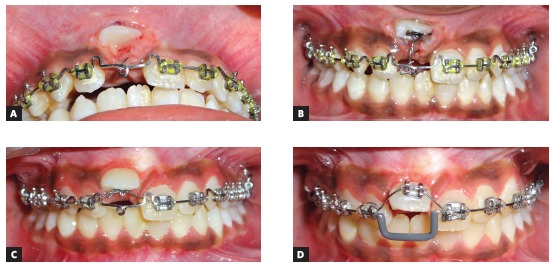
A) Surgically exposed palatal surface of impacted #11 using window excision of soft tissues B) Begg’s bracket bonded on palatal aspect of #11, accompanied by initiation of forced eruption. C) Progression of orthodontic traction. D) Preadjusted Edgewise bracket bonded on labial aspect and continuation of traction using piggyback 0.014-in improved superelastic NiTi archwire over 0.019 x 0.025-in stabilizing base archwire.

A pre-adjusted Edgewise appliance (MBT prescription, 0.022 x 0.028-in slot) was placed in the maxillary arch. Initial alignment and leveling was achieved with super-elastic 0.016-in nickel-titanium (NiTi) wire. After adequate space creation using a compressed nickel-titanium open-coil spring between right maxillary lateral incisor a nd left maxillary central incisor on a 0.016 x 0.022-in stainless steel (SS) wire, the patient was referred to the oral surgeon for surgical exposure of the dilacerated central incisor. Under local anesthesia, a window was created to expose the tooth crown (Fig 4A). Minimal elevation of the overlying mucoperiosteum and follicle of the lingual surface was done just enough to allow bonding a Begg’s bracket on the uncovered palatal surface. The alveolar bone layer and connective tissue follicle of the labial surface was retained to permit hemostasis. A 0.019 x 0.025-in SS wire with bilateral ‘V’ bends (for effective anterior and posterior torque control) and vertical stops abutting the mesial aspects of the right maxillary lateral incisor and left maxillary central incisor was used as a main stabilizing archwire. Orthodontic traction was initiated using light orthodontic force (≈ 40g) with a 0.010-in SS ligature wire tied to the main archwire (Fig 4B). As the tooth responded to the force, it rotated downwards as it migrated occlusally (Fig 4C). 

Traction continued using elastic threads which were changed every three weeks. After five months, when the labial surface was sufficiently visible, an orthodontic bracket was bonded on the labial surface to permit palatal movement of the crown. Further alignment progressed by placing improved super-elastic 0.014-in NiTi wire piggyback over a 0.019 x 0.025-in SS base wire, which in turn prevented any arch deformation due to reactionary forces (Fig 4D). Simultaneously, preadjusted Edgewise appliance was placed in the mandibular arch.

**Figure 5:**
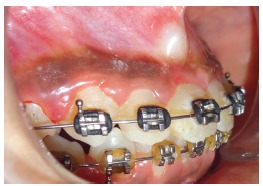
Intraoral view showing the labial radicular bulge of #11 after orthodontic alignment.

**Figure 6:**
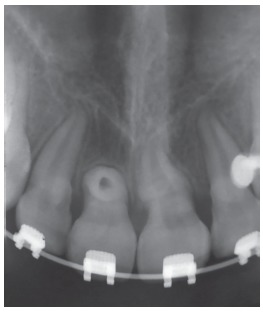
Post-alignment IOPA x-ray showing the characteristic “bull’s-eye” appearance of the dilacerated root.

Once aligned, root apex of the right maxillary central incisor could be palpated just below the labial alveolar mucosa and the patient complained of discomfort in the region and found it esthetically displeasing (Fig 5). Periapical radiograph demonstrated a “bull’s eye” appearance characterized by rounded opaque area with a dark spot in its center, caused by the apical foramen of the root canal of the dilacerated apical portion of root (Fig 6). The periodontal ligament space around the dilacerated portion of the root was evident as a radiolucent halo, with increased radiopacity of the dilacerated segment, compared to the rest of the root, due to the increased thickness of tooth structure that the x-rays had to pass through.

**Figure 7:**
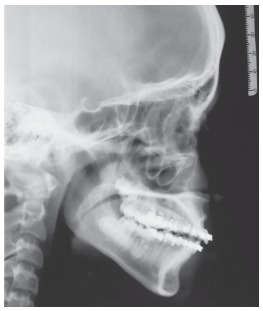
Post-endodontic lateral cephalogram depicting well-obturated dilacerated portion of the root.

**Figure 8:**
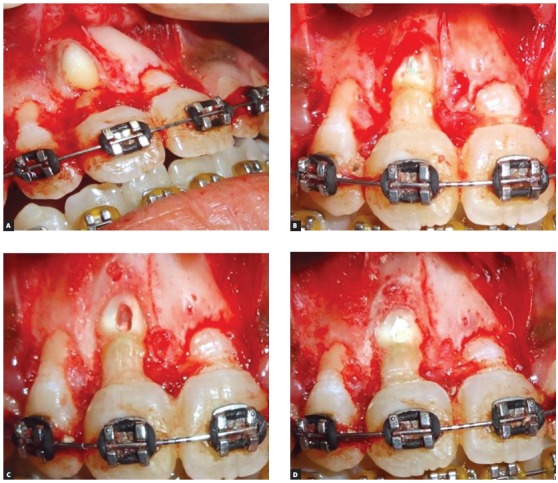
A) Bony fenestration observed over the labial bulge of the apical aspect of the root. B) After adequate trimming of the labial bulge. C) Cavity prepared on trimmed labial portion of the root. D) Cavity restored with biodentine.

Consultation was sought from the endodontist and root canal treatment was planned followed by apicoectomy with retrograde root filling. Rubber dam was set using the split dam technique. Opening access was made and #6, 8, 10 K-files (C+ files, Dentsply, Maillefer, Ballaigues, Switzerland) were used to negotiate the canal up to the apex. The canal was prepared using NiTi files (#15 to #40). Once the canal had been sufficiently debrided, the root canal was obturated using injectable gutta-percha 3D obturation (Calamus dual, Dentsply, Tulsa Dental, TN, USA) system. The lateral cephalogram revealed labial projection of apical third of the root (Fig 7) and an apicoectomy was scheduled. Once the full-thickness rectangular mucoperiosteal flap was reflected, a bony fenestration was observed over the labial bulge of the apical third of the root (Fig 8A). After adequate trimming of the labial bulge (Fig 8B), the flap was temporary repositioned to ensure that the bulge was no longer palpable. Using an inverted cone bur, a cavity was prepared on the trimmed labial portion of the root (Fig 8C). The cavity was restored with biodentine (Fig 8D), and thereafter the flap was approximated and sutured back in its original position.

Following endodontic therapy, a window period of two months was given to ensure that the patient was completely asymptomatic before orthodontic treatment could be resumed. Then, 0.019 x 0.025-in beta-titanium (TMA) wire with tie-back was used during finishing stage, for improving torque in maxillary incisor region. After settling and detailing of occlusion, the fixed appliances were debonded and wraparound retainers were placed in maxillary and mandibular arches.

## TREATMENT RESULTS

**Figure 9:**
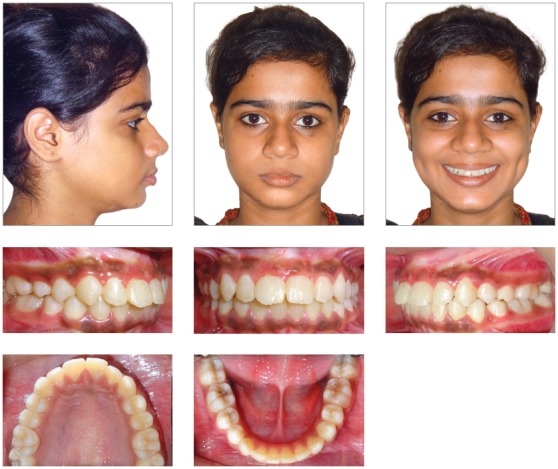
Posttreatment photographs.

**Figure 10:**
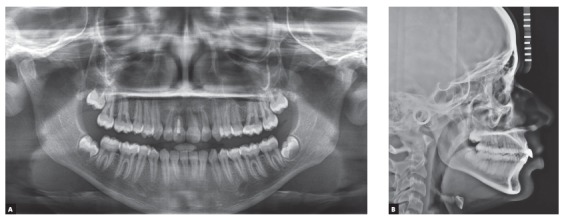
A) Posttreatment panoramic radiograph. B) Posttreatment lateral cephalogram.

Through a meticulous interdisciplinary orthodontic and endodontic approach, the impacted right maxillary central incisor was brought into normal occlusion following 15 months of treatment. When compared with contralateral central incisor, no clinically significant differences were observed in the clinical crown length and probing depth measured with a standard periodontal probe at the mesiolabial, midlabial, distolabial, mesiopalatal, midpalatal and distopalatal surfaces of each tooth. Frontal facial esthetics showed significant improvement, with the repositioned incisor displaying acceptable gingival contour and width of attached gingiva (Fig 9). The posttreatment radiographs demonstrated good periodontal health, with well aligned near normal shape of the root of corrected incisor (Fig 10). 

**Figure 11:**
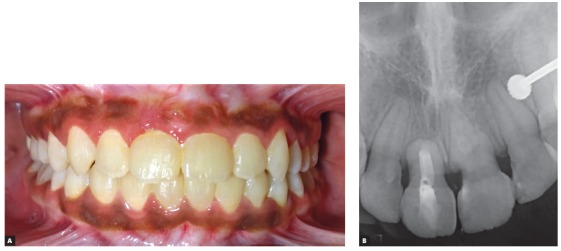
A) Intraoral frontal view at 3-year follow up. B) Periapical radiograph at 3-year follow up.

Based on a 5-point categorical visual analog scale (i.e. ‘dissatisfied’, ‘partially satisfied’, ‘mostly satisfied’, ‘pleased’ and ‘delighted’), the patient was delighted with the improvement in facial and smile esthetics. At three-year follow-up, the incisor showed good orthodontic and periodontal stability, without any evidence of root resorption (Fig 11).

## DISCUSSION

The orthodontic alignment of an impacted maxillary incisor with severe root dilaceration in labial direction is an arduous clinical task. Various factors that govern the successful alignment of such impacted dilacerated teeth include:^12^ ^,^ ^13^

» position and direction of the impacted tooth;
» extent of root formation;
» direction and angulation of dilaceration;
» amount of space available for aligning the impacted tooth.

The most frequently reported orientation of dilacerated maxillary incisors is an upward and labial coronal inclination, which presents with good to fair prognosis. On the other hand, dilacerated teeth with the crown in an inverted orientation are known to have a doubtful prognosis. Factors governing favorable prognosis for orthodontic traction of a dilacerated tooth include an obtuse inclination angle, incomplete root formation and a lower position in relation to the alveolar crest.^8^ ^,^ ^14^

Since conventional radiographic views such as periapical, cephalometric and panoramic X-rays provide insufficient two-dimensional information, CBCT imaging proves to be a valuable diagnostic aid in such cases by enabling further localization and assessment of the dilacerated impacted tooth. Thus, in the reported case, three-dimensional reconstructions were used to ascertain the accurate position of the impacted tooth, its proximity to adjacent tooth, stage of root formation and extent of curvature and direction of the dilacerated root in three dimensions. Based on sagittal section revelations of three-fourth root formation, i.e. stage 8 of Nolla’s classification,^15^ the impacted incisor was assigned to the early dental-age group; whereas its counterpart with an almost complete root but an open apex (stage 9) was allocated to the late dental-age group. The presence of unfavorable crown/root proportion along with dilacerated root facing superiorly upwards and positioned at a higher level in the alveolar bone posed a clinical challenge.

The etiology of dilacerations is usually ascribed to a mechanical traumatic injury to the calcified portion of a developing tooth in the deciduous dentition. Acute traumatic injury involving intrusion or avulsion of overlying deciduous tooth before four years of age usually results in dilaceration of the underlying developing succedaneous permanent tooth.^16^ As no evidence of trauma could be elicited, idiopathic developmental disturbance could be attributed as the cause of dilaceration in the present case. As theorized by Sun et al,^13^ labial rotation of the crown bringing the Hertwig’s epithelial root sheath in close proximity to the palatal cortical bone might have resulted in impeded space for root development.

## SURGICAL CONSIDERATIONS

Appropriate surgical management of impacted dilacerated teeth is vital for obtaining desirable esthetic gingival outcomes. Surgical exposure of such impacted teeth may be carried out using three different techniques, namely: the window excision of soft tissues, an apically positioned flap or a closed eruption technique.

Surgical exposure by window excision of soft tissues was planned as the procedure of choice in the present case to gain access to the palatal surface of the impacted incisor, since the bulge was high in the sulcus. An apically positioned flap, which has been recommended to preserve the keratinized tissue, was not used due to the highly positioned impacted incisor. In accordance with the recommendations of Bishara et al,^17^ a conservative exposure of the impacted incisor was performed to allow for the placement of a bonded Begg’s bracket. Similar to the approach of Uematsu et al^6^, the labial epithelial attachment on the impacted incisor was retained so that the repositioned incisor would present acceptable gingival contour and attached gingiva. The disadvantage of gingival scarring or increase in clinical crown length associated with the window approach, as has been reported by Boyd et al,^18^ was not observed in the present case as very light forces were employed for protracting the maxillary right central incisor into proper alignment in the arch.

It has been reported that even a dilacerated tooth with root completion about one-third to one-half its normal length can restore facial esthetics and maintain adequate alveolar bone support.^19^ However, due to the chances of root apices coming in contact with the labial cortical plates during torque control, dilacerated roots are more susceptible to exposure and resorption, and this needs to be discussed with the patients beforehand. In the present case, stability was well maintained three years after treatment, with the dilacerated root showing no apparent resorption, probably because of cementum repair. 

## ENDODONTIC CONSIDERATIONS

Endodontic therapy was sought necessary post incisor alignment since the root apex demonstrated a bulge just beneath the labial alveolar mucosa, and the patient was concerned about the unaesthetic appearance. 

The split rubber dam technique, as used in this case, allowed adequate isolation in the presence of orthodontic brackets, where the placement of rubber dam by the conventional method was otherwise not feasible. Since a “scout file” provides critical information regarding the extent and direction of root canal dilacerations, it was used before initiating shaping procedures. In such cases, precurving of all endodontic instruments (especially those larger than size 20) is mandatory, in order to allow the files to follow the curvature of the canal without cutting in a straight direction. Moreover, the instruments should be considered as “single use instruments”. 

The outcome of root canal treatment in dilacerated teeth is mainly dependent on complete biomechanical preparation and elimination of microorganisms from the root canal system. A multi-visit approach involving frequent repetitive copious irrigation and file recapitulation in conjunction with the use of interappointment intracanal medicaments has shown to improve the predictability of root canal treatment in such teeth.^20^

Calcium hydroxide and Triple Antibiotic Paste (combination of metronidazole, ciprofloxacin, and minocycline) are frequently used intracanal medicaments to help disinfect the root canal system. Calcium hydroxide mixed with glycerin rather than sterile water has shown significantly superior results with regards to the length of filling and density in the apical third of curved canals. 

When using lateral compaction technique, the arc of movement of NiTi spreaders in dilacerated canals should be less than 90^o^ as compared to 180^o^ in non-dilacerated canals.^21^ Although technique sensitive, the use of warm or thermoplasticized gutta-percha might also be beneficial in some cases.

The removal of a portion of the dilacerated root using the apicoectomy technique has been advocated in some cases following orthodontic traction.^6^ ^,^ ^7^ Following successful traction of the impacted right maxillary central incisor into proper alignment, the curved root apex abutted the labial cortical plate and the radicular bulge was palpable under the soft tissue, which necessitated root canal treatment accompanied by an apicoectomy for removal of dilacerated root-end.

## CONCLUSION

The orthodontic alignment of a severely dilacerated impacted incisor represents a challenging clinical scenario. Meticulous and precise treatment planning considerations, involving the interdisciplinary communicative feedback between the oral surgeon, orthodontist and endodontist helped achieving successful esthetic, structural and functional outcome in the present case. As is the case with any treatment, the results are rewarding if priority is given to the patient’s concerns and expectations, at the same time ensuring the overall well-being of the patient.

## References

[1] Andreasen JO, Sndström B, Ravn JJ (1971). The effect of traumatic injuries to primary teeth on their permanent successors A clinical and histologic study of 117injured permanent teeth. Scand J Dent Res.

[2] Zilberman Y, Ben Bassat Y, Lustmann J, Fuks A, Lustmann J (1986). Effect of trauma to primary incisors on root development of their permanent successors. Pediatr Dent.

[3] Sakai VT, Moretti AB, Oliveira TM, Silva TC, Abdo RC, Santos CF (2008). Replantation of an avulsed maxillary primary central incisor and management of dilaceration as a sequel on the permanent successor. Dent Traumatol.

[4] Tsai TP (2002). Surgical repositioning of an impacted dilacerated incisor in mixed dentition. J Am Dent Assoc.

[5] Maia RL, Vieira AP (2005). Auto-transplantation of central incisor with root dilaceration Technical note. Int J Oral Maxillofac Surg.

[6] Uematsu S, Uematsu T, Furusawa K, Deguchi T, Kurihara (2004). Orthodontic treatment of an impacted dilacerated maxillary central incisor combined with surgical exposure and apicoectomy. Angle Orthod.

[7] Valladares J, Costa SP, Estrela C (2010). Orthodontic-surgical-endodontic management of unerupted maxillary central incisor with distoangular root dilaceration. J Endod.

[8] Pavlidis D, Daratsianos N, Jäger A (2011). Treatment of an impacted dilacerated maxillary central incisor. Am J Orthod Dentofacial Orthop.

[9] Wei YJ, Lin YC, Kaung SS, Yang SF, Lee SY, Lai YL (2012). Esthetic periodontal surgery for impacted dilacerated maxillary central incisors. Am J Orthod Dentofacial Orthop.

[10] Celli D, Greco AL, Sferra S, Deli R (2015). Management of impacted dilacerated maxillary incisor with strategic positioning of a straightwire appliance. Eur J Paediatr Dent.

[11] Chang NY, Park JH, Kim SC, Kang KH, Cho JH, Cho JW (2016). Forced eruption of impacted maxillary central incisors with severely dilacerated roots. Am J Orthod Dentofacial Orthop.

[12] Topouzelis N, Tsaousoglou P, Pisoka V, Zouloumis L (2010). Dilaceration of maxillary central incisor a literature review. Dent Traumatol.

[13] Sun H, Wang Y, Sun C, Ye Q, Dai W, Wang X (2014). Root morphology and development of labial inversely impacted maxillary central incisors in the mixed dentition a retrospective cone beam computed tomography study. Am J Orthod Dentofacial Orthop.

[14] Andrade MG, Weissman R, Oliveira MG, Heitz C (2007). Tooth displacement and root dilaceration after trauma to primary predecessor an evaluation by computed tomography. Dent Traumatol.

[15] Nolla C (1960). The development of the permanent teeth. J Dent Child.

[16] Colak I, Markovic D, Petrovic B, Peric T, Milenkovic A (2009). A retrospective study of intrusive injuries in primary dentition. Dent Traumatol.

[17] Bishara SE (1992). Impacted maxillary canines a review. Am J Orthod Dentofacial Orthop.

[18] Boyd RL (1984). Clinical assessment of injuries in orthodontic movement of impacted teeth II. Surgical recommendations. Am J Orthod.

[19] Lei L, Yan F, Li H, Li H (2015). Treatment of dilacerated Incisors in early and late stages of root development. J Clin Orthod.

[20] Jafarzadeh H, Abbott PV (2007). Dilaceration: review of an endodontic challenge. J Endod.

[21] Cohen S, Burns RC (2002). Pathways of the pulp.

